# Supplementary material for: GhMAX2 Contributes to Auxin-Mediated Fiber Elongation in Cotton (Gossypium hirsutum)
Source: Plants (Basel). 2024 Jul 25;13(15):2041. doi: 10.3390/plants13152041 (PMC11314591; doi:10.3390/plants13152041)
Supplement: Supplementary file 1 [file plants-13-02041-s001.zip › Supplementary Figure.pdf]

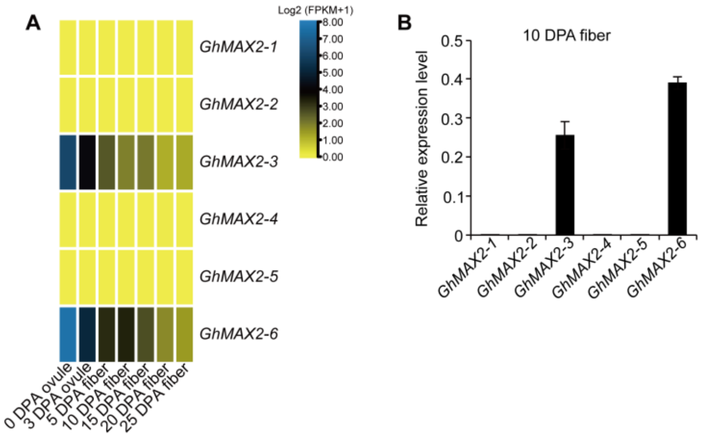

Figure S1. Heatmap and RT-qPCR showing the relative expression level of *GhMAX2* in cotton fibers based on the RNA-seq data. The transcriptome data was from NCBI under the project number PRJNA634606.

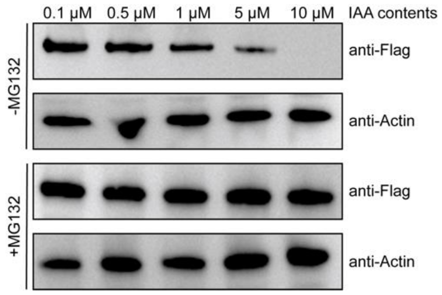

Figure S2. In vivo degradation assay showing that IAA promotes GhIAA17 degradation in a dose-dependent. *GhIAA17-Flag* construct was transfected into cotton protoplasts and cultured for 12 h in dark. The protoplasts were then treated with different concentrations of IAA and 200 mM CHX with or with or without 5 μM MG132 for 2 h. Total proteins were extracted and subjected to western blotting analysis using anti-Flag and anti-Actin antibodies.

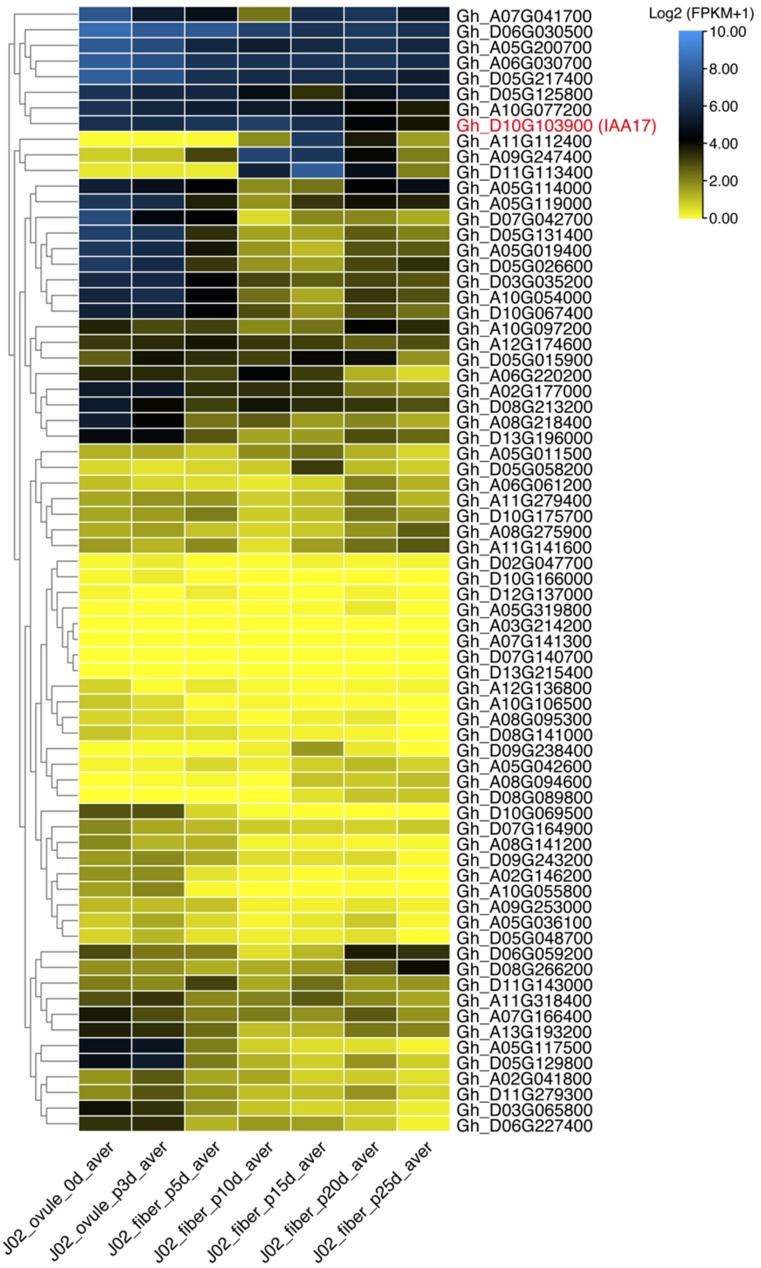

Figure S3. Heatmap showing the expression level of *GhIAA* family genes in cotton fibers based on the RNA-seq data. The transcriptome data was from NCBI under the project number PRJNA634606.

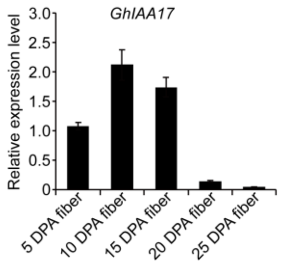

Figure S4. RT-qPCR analysis of GhIAA17 transcript level in cotton fibers.
