# Supplementary material for: GhMAX2 Contributes to Auxin-Mediated Fiber Elongation in Cotton (Gossypium hirsutum)
Source: Plants (Basel). 2024 Jul 25;13(15):2041. doi: 10.3390/plants13152041 (PMC11314591; doi:10.3390/plants13152041)
Supplement: Supplementary file 1 [file plants-13-02041-s001.zip › Supplementary Table.pdf]

Table S1. GhMAX2-3 interaction protein candidates by Y2H screening

ID	Annotation
Gh_A01G007300	EKC/KEOPS complex subunit tprkb-like protein
Gh_A01G007900	Leucine-rich repeat (LRR) family protein, LRX4
Gh_A01G008300	DNA-DAMAGE REPAIR/TOLERATION 100, DRT100
Gh_D10G103900	INDOLE-3-ACETIC ACID INDUCIBLE 17, IAA17
Gh_A01G009300	Ribosomal protein S14p/S29e family protein, US14X
Gh_A05G051300	COP9 SIGNALOSOME 5A, CSN5A

Table S2. Primers used in this study

Primer name	Application	Sequence (5'-3')
MAX2-3-F	RT-qPCR	GTCAGCCGTAAGTTTATGCT
MAX2-3-R	RT-qPCR	AGGAAGGAGAGGTCCAAGTC
MAX2-6-F	RT-qPCR	CCTGCGGTCACGTCCTTGAC
MAX2-6-R	RT-qPCR	GTCTTCACCGATTGGCCACG
IAA17-F	RT-qPCR	CATATACACTGAAAAAAAAT
IAA17-R	RT-qPCR	TTTGGTTCCATTACTGGTGC
MAX2-3-BD-F	Y2H	GGCCATGGAGGCCGAATTCATGGCCA CCATTAACGATATCCCCGACGT
MAX2-3-BD-R	Y2H	TCGACGGATCCCCGGGAATTCTCAATC GAGGATGGGTCGCC
MAX2-6-BD-F	Y2H	ATGGCCATGGAGGCCGAATTCATGGCC ACCATTAACGATATCCC
MAX2-6-BD-R	Y2H	TCGACGGATCCCCGGGAATTCTCAATC GAGGATGTGTCGCC
IAA17-AD-F	Y2H	GCCATGGAGGCCAGTGAATTCATGTCT CCGCTGTTGCTTGG
IAA17-AD-R	Y2H	ATTCATCTGCAGCTCGAGCTCCTAACA GTCTTTCTTGGACT
MAX2-3-cLUC-F	LCI	ATGGCCATGGAGGCCGAATTCATGGCC ACCATTAACGATAT
MAX2-3-cLUC-R	LCI	acgaaagctctgcaggtcgacTCAATCGAGGAT GGGTCGCC
MAX2-6-cLUC-F	LCI	acgaaagctctgcaggtcgacTCAATCGAGGAT GTGTCGCC
MAX2-6-cLUC-R	LCI	tacgcgtcccggggcggtaccATGGCCACCATT AACGATAT
IAA17-nLUC-F	LCI	cgagctcggtacccgggatccATGTCTCCGCTG TTGCTTGG
IAA17-nLUC-R	LCI	cgcgtacgagatctggtcgacACAGTCTTTCTT GGACTTGT
IAA17-Flag-F	Y2H	CAAATCGACTCTAGAAAGCTTATGTCTC CGCTGTTGCTTGG
IAA17-Flag-R	Y2H	GTCTTTGTAGTCCATAAGCTTACAGTCT TTCTTGGACTTGT
MAX2-3-Myc-F	Protein extraction	ACTTGAACTCGGTAgagctcATGGCCACC ATTAACGATATC
MAX2-3-Myc-R	Protein extraction	cgatcggggaaattcGAGCTCTCAATCGAGG ATGGGTCGCC
MAX2-6-Myc-F	Protein extraction	GACTTGAACTCGGTAgagctcATGGCCAC CATTAACGATAT
MAX2-6-Myc-R	Protein extraction	cgatcggggaaattcGAGCTCTCAATCGAGG ATGTGTCGCC

IAA17-Myc-F	Protein extraction	cgatcggggaaattcGAGCTCCTAACAGTCTTTCTTGGACT
IAA17-Myc-R	Protein extraction	GACTTGAACTCGGTAgagctcATGTCTCCGCTGTTGCTTGG
MAX2-3-GFP-F	Protein extraction	CGGGGGACtctagaggatccATGGCCACCATTAACGATATC
MAX2-3-GFP-R	Protein extraction	GCCCTTGCTCACCATGGATCCATCGAGGATGGGTCGCCTAT
MAX2-6-GFP-F	Protein extraction	ACGGGGGACtctagaggatccATGGCCACCATTAACGATAT
MAX2-6 -GFP-R	Protein extraction	GCCCTTGCTCACCATGGATCCATCGAGGATGTGTCGCCTAT
MAX2-3-VIGS-F	VIGS	AAGCTTATGGCCACCATTAACGATAT
MAX2-3-VIGS-R	VIGS	TGCGGAGGCGATGGGCCAAAGAATTC
MAX2-6-VIGS-F	VIGS	AAGCTTTAAATTGACGCTTTTGCATT
MAX2-6-VIGS-R	VIGS	ACTGCCCCAACTTGAGCACTGAATTC
IAA17-VIGS-F	VIGS	AAGCTTAACTTGATGAGAAGACACTG
IAA17-VIGS-R	VIGS	TCTTGTATCTCATTTGCAGGGAATTC
